# New Measure of Economic Development Based on the Four-Colour Theorem

**DOI:** 10.3390/e23010061

**Published:** 2020-12-31

**Authors:** Aleksander Jakimowicz, Daniel Rzeczkowski

**Affiliations:** 1 Department of World Economy, Institute of Economics, Polish Academy of Sciences, Palace of Culture and Science, 1 Defilad Sq., 00-901 Warsaw, Poland; 2 Department of Market and Consumption, Faculty of Economic Sciences, University of Warmia and Mazury in Olsztyn, 1/327 Cieszyński Sq., 10-720 Olsztyn, Poland; daniel.rzeczkowski@uwm.edu.pl

**Keywords:** measure of economic development, websites, public administration sector, municipality, four-colour theorem, prosumption, platforms for participation, location quotient, dual graph, Euler characteristic

## Abstract

The location quotient is one of the basic quantitative tools for identifying the regional poles and the turnpikes of economic growth in spatial economy. The disadvantage of this traditional measure is the limited scope of economic information contained in it. The new measure of economic development proposed in the article encompasses a complex spectrum of phenomena in one number, as it takes into account the influence of the public administration sector, as well as top technology in the form of ICT and its practical business models. It also takes into account the digital prosumption and the platforms for participation. The participation platforms in the public administration sector are the websites of municipal public administration offices. A cluster analysis was used to distinguish four quality classes of these websites. These classes were assigned four different colours, which were then used to draw up a map of the selected province. Each municipality is marked with a colour that corresponds to the quality class of the website of the state administration office operating on its territory. The colour system resulting from the four-colour theorem and the corresponding dual graph play the role of a reference system in relation to each empirical colour distribution and another dual graph related to it. The measure of the economic development of a region is the degree of reduction of the dual graph corresponding to the empirical distribution of colours, which identifies the actual growth poles and determines the routes of growth. The presented indicator better and more precisely identifies poles and routes of economic growth than the traditional location quotient.

## 1. Introduction

Development of new technologies has an enormous impact on economic growth. John Hicks in the classic work *Capital and Growth* notes that each production technique has a corresponding rate of return, which is fully determined only by this technique [1]. If it is possible to choose a production technique, then there should be a technique with the highest rate of return, achievable along a growth equilibrium path, within the technology. Such a technique is labelled as top technique. It corresponds to the maximum rate of sustainable growth in the known John von Neumann model [2]. Contemporarily, this role is played by the digital technique, which transformed the traditional economy into the digital economy. Therefore, its inclusion is necessary in all studies on economic growth and development.

The impact of digital computing technologies on the economy is at the centre of the dynamically developing research trend called wikinomics [3,4]. It was established that there was a significant change in business rules, which led to the creation of completely new business models. The most important of them include prosumption and participation platforms. The first implies active involvement of consumers (recipients) who are a non-standard source of innovation and creativity, in the design and manufacture of new products (services) [5,6]. Participation platforms include products and technology infrastructure made available to large communities of partners. Access to these products and technologies enables the creation of new values and initiates innovative ventures. Prosumption is increasingly becoming digital and participation platforms are a medium for the flow of economic information.

Participation platforms take the form of websites that help enterprises collaborate with business partners, clients and the wider economic environment. They enable practical implementation of such principles of wikonomics as openness, peering, sharing, and acting globally. Participation platforms in the private sector have become the engine of economic development, as they have contributed to the increase in production capacity without the need of incurring additional fixed costs. In addition, the creation of open participation platforms greatly facilitates innovation activities. Web services mashups are becoming increasingly popular, as they combine various ready-made services or applications, made available by other websites, into a new whole which has improved quality and functionality.

The possibilities of using participation platforms are virtually limitless, they can be the basis for development for many different products or services, literally everything that can be controlled by software. Therefore, participation platforms are not limited to commercial applications, but can also perform many useful functions in the public administration sector. There are large databases in this sector that are hardly used. They could become a source of many new services stimulating economic growth and development. Thus, societies do not make full use of all the opportunities resulting from technical progress to improve their standard of living. Websites in public administration should take the form of platforms for grassroots action and include platforms for public disclosure and platforms for neighbourhood knowledge [3] (pp. 199–205).

The purpose of the article is to develop a new, synthetic measure of economic growth in the regional perspective, which would include in one number abundant information relevant from the point of view of local entrepreneurship. The object of research is the Warmia and Mazury Province, consisting of 116 municipalities, located in north-eastern Poland. This province is one of the least developed in Poland [7,8], but in recent years many economic initiatives have been undertaken in order to accelerate the development and reduce the distance to the rest of the country [9,10]. The proposed measure meets Hicks’s condition for top technique because it is based on wikinomics business models in the form of prosumption and participation platforms which are characteristic for digital economy. In addition, this measure considers the spatial diversity of municipalities, the barriers they encounter in mutual economic cooperation, and it is also useful for locating regional poles and turnpikes of growth. As it is based on digital technologies, it is not limited to one industry, effectively covers all types of economic activities that can be controlled by software. This is an advantage over classic measures of local growth and development like the location quotient.

Currently, websites of municipal public administration offices—basic units of the local government in Poland—are much less developed than platforms for commerce, which means that not all factors of regional economic growth are available to municipal communities. From the point of view of wikinomics, these websites should be treated as the basic sources of entrepreneurship, as their role should be to initiate and develop business activities in municipalities. Empirical research confirms the impact of the size of local government administration at a municipal level on entrepreneurship, but this impact is not clear. It has been observed that the increase in the size of administration affects entrepreneurship in a negative way [11]. This justifies the need to observe this issue from the digital technique point of view. Therefore, the starting point of the study was to assess the functionality of websites of municipal public administration offices. The evaluation criterion was the degree of their fulfilment of the role of wikinomics platforms for participation. By means of cluster analysis, these websites were grouped into four distinct quality classes: low, medium, high and very high. Four different colours were assigned to these classes, which were used to map the evaluated province. Each municipality was marked with a colour corresponding to the quality class of its website. As the map of the studied region is a normal map, it meets the conditions of the four-colour theorem. In this way, two types of colour systems can be distinguished. The first results from the application of the theorem itself, while the second includes empirical colour distributions reflecting the quality classes of websites in specific periods. Based on each map, a corresponding dual graph was prepared. Capitals of all municipalities were marked on the map of the province, and then the capitals of the neighbouring municipalities were connected by roads crossing their common borders. The dual graph meeting the conditions of the four-colour theorem is a reference frame for the research. On empirical maps, the edges of the graph, indicate barriers to cooperation between individual municipalities. The new measure of economic growth is determined by comparing the number of edges of empirical graphs with the reference graph. For this purpose, the generalisation of the Euler characteristic made by Augustin-Louis Cauchy was used. In addition, the article discusses the problem of dividing websites into four quality classes in the light of René Thom’s classification theorem. The impact of the complexity of geographical lines forming boundaries between municipalities on possibilities of application of the four-colour theorem is also being studied. It can be observed that municipalities in the Warmia and Mazury Province are fractals, similar to natural structures such as coastlines or clouds and the four-colour theorem can be used to describe infinitely complex fractal structures.

This paper precisely defines all the methods used in the research to accurately identify the sources of complexity emerging in the examined system and to facilitate their economic description. The research belongs to complexity economics, which implies that it is also an element of a wider discipline—the science of complexity. Advances in information technology have contributed to significant changes in economic life and have resulted in the need to modify classic economic laws and to discover new ones. This study combines several mathematical, physical and economic methods applied to obtain an overall measure of the economic performance of any region consisting of sub-units, which would allow determining growth and development based on the rules of wikinomics and, in the same time, take into account top technology as understood by Hicks. The four-colour theorem plays an essential role in the measure of development proposed in the paper. Number four is not a random number, but an attractor of the economic system, as indicated by the catastrophe theory formulated by René Thom. The subject of the research is the dynamics of objects in three-dimensional physical space–time, which consists of two spatial dimensions and one temporal dimension. These objects are dual graphs based on five maps of the examined province, one of which was coloured according to the four-colour theorem principles and provides a system of reference, while the other four are of an empirical nature and present the distribution of website quality classes of all municipalities in the four periods under consideration. All events taking place in this space–time continuum consist of the transformations of two-dimensional empirical dual graphs over time. What does not change is only the dual graph corresponding to the map of the province, coloured according to the four-colour theorem principles. Following the classification theorem, which underlies catastrophe theory, in three-dimensional physical space–time there are only five elementary catastrophes, which represent all possible manifestations of dynamic phenomena. Therefore, all empirical distributions of the municipality websites quality classes must coincide with four elementary catastrophes, while the fifth catastrophe represents the dynamics of the environment, i.e., the rest of the national economy since the examined province is an open system. Consequently, the empirical division of municipality websites into four quality classes, resulting from the use of the *k*-means algorithm, is correct in a topological sense. In addition, the province of Warmia and Mazury—comprising one hundred and sixteen municipalities—is a system so diverse that it provides an excellent research subject ensuring an unbiased test verifying the correctness of the presented method for economic growth measurement. In other words, this province is complex enough to allow empirical demonstration of the attractor in the form of the number four. It is also possible to note significant mathematical links between the catastrophe theory and the four-colour theorem. They lead to the conclusion that the classification theorem provides topological proof of the four-colour theorem. This fact has not been recognised in science so far and we are probably the first researchers to discover it.

If two adjacent municipalities have websites marked with different colours, i.e., belonging to different quality classes, then the edge crossing the common border and connecting their capitals symbolises the absence of significant economic cooperation between them. The existence of cooperation would mean the unification of websites and their transfer to high or very high quality classes. On the other hand, if neighbouring municipalities have websites marked with the same colours and therefore belong to the same quality class, the absence of edges represents economic cooperation between them. The four-colour theorem is the reference system for the research since the resulting dual graph represents the total absence of growth poles and cooperation between municipalities. In this case, every two adjacent municipalities are connected by edges. For Warmia and Mazury, the number of these edges determined based on Cauchy’s theorem is 277. This value always appears in the denominator of Equation (16), which defines the proposed measure of economic development, while the numerator contains the changing numbers of the edges of the empirical dual graphs determined for each year, i.e., 63, 49, 106 and 58. Thus, the defined measure is an absolute measure, which makes it more objective compared to relative measures commonly used in economics (e.g., the method for calculating real national income based on price deflators).

The methodological procedure used to develop a new measure of economic development is complex as it is based on a combination of many different approaches. Its individual stages can be summarised in the following points:

1. Selection of the province for which the measure is to be designated. Such a region should be sufficiently diversified economically and it should consist of a large number of municipalities.
2. Development of qualitative criteria for the evaluation of municipal websites on the basis of wikinomics guidelines.
3. Binary evaluation of websites, which consists in assigning each website the value of one for meeting a given criterion. If the specified criterion is not met, the assigned value is zero.
4. Calculation of the quality indicator for each evaluated website according to the sum of the values received for the fulfilled criteria. The maximum number of points signifies that all qualitative criteria are met.
5. Division of websites into homogeneous subsets called quality classes with the use of the *k*-means clustering method. According to the classification theorem by René Thom, the attractor of this division is the number four, provided the region is sufficiently differentiated.
6. Assignment of colour to each quality class and preparation of an empirical map of the province with the inclusion of the colour coding of the municipalities according to the quality classes of their websites.
7. Preparation of an absolute reference system in the form of a map of the province coloured according to the four-colour theorem.
8. Preparation of the dual graphs for the empirical maps and reference map. Comparison of the number of edges of the empirical dual graph with the number of edges of the reference dual graph is the essence of the proposed measure of economic development. The edges of the graphs represent the lack of economic cooperation between neighbouring municipalities.

The evaluation of municipalities on the basis of the quality of the websites is not the only way to determine the proposed measure of economic development. This measure may be of a more general nature. It could be based on a different socio–geographic–economic indicator that would apply to all municipalities in a given province and would have four elements in the internal structure.

## 2. The Location Quotient

The location quotient is one of the basic quantitative tools used to identify regional poles and turnpikes of growth in spatial economy. It is used to compare two spatial units described by percentage indicators, one of which refers to the characteristics of a given region (municipality, province), while the other refers to a higher spatial unit (province or country). The location quotient measures the region’s industrial specialisation in relation to a larger geographic unit [12,13,14]. Any base that is significant for the problem and region under study can be used. For this reason, the location quotient can find application even in sciences such as criminology [15]. Sometimes bases such as earnings or GDP by metropolitan area are used, however employment is most often accepted as the most relevant base. Employment in various sectors of industry is distinguished, moreover trade and services are also included. The location quotient $\left(LQ_{i_{n}}\right)$ is given by [16,17]:

(1) LQin=EinETn∑n=1NEin∑n=1NETn,

where: n—small area under study, N—total number of areas, $E_{i}$—employment in industry i, and $E_{T}$—total employment in all industries.

The location quotient is used in economic research as in [18] (pp. 173–176):

(1) a measure of relative concentration or location advantage/disadvantage of specific industries in the regional economy;
(2) a proxy for input–output coefficients used to assess regional or subregional multiplier effects.

As a measure of relative concentration, it demonstrates the strengths and weaknesses of various industries in the region. If the location quotient for the *i*-th industry is greater than one, then the region is assumed to be exporting the products or services of that industry. Then, in a given area, relatively more employees are employed in a specified sector of industry compared to a larger area which implies that the given area produces more goods or services than are consumed by the area’s residents. The excess can therefore be exported. When the location quotient is less than or equal to unity, the industry does not export outside the region, because it is not self-sustainable. In other words, Equation (1) shows whether the region is an exporter or importer of goods produced by the *i*-th industry. For $LQ_{i_{n}}>1$ the export activity $\left(X_{i_{n}}\right)$ of regional employment in i-th industry $\left(E_{i_{n}}\right)$ is calculated as follows [16]:

(2) Xin=[1−(1LQin)]Ein.

Multiplier effects appear due to the fact that a change in regional export activity leads to a total change in the regional economy. The economic base multiplier is greater than one by the proportion of local to export activity. It is a special case of the traditional Keynesian multipliers [19]. A total change in the regional economy is a product of the multiplier and the export change. The economic base multiplier can be determined from the location quotient as follows [16]:

(3) $$X_{i_{n}}=\left(\frac{E_{i_{n}}}{E_{T_{n}}}-\frac{\sum_{n=1}^{N}E_{i_{n}}}{\sum_{n=1}^{N}E_{T_{n}}}\right)E_{T_{n}}.$$

Typically, the economic base multiplier is calculated by determining the export employment on the basis of Equation (2) for all industries for which $LQ_{i_{n}}>1$, by summing the export employment of all those industries, and dividing the sum by total employment. Formula (3) shows that this traditional calculation process can be simplified. The calculation of export employment involves subtracting the share of the nation’s economy accounted for by *i*-th industry from the share of the region’s economy accounted for by *i*-th industry, and the resulting difference is multiplied by the region’s total employment.

This article examines municipalities as the basic spatial units. The superior unit is a province and, therefore, interpretation of the Formula (1) is affected. If we understand small areas as municipalities in the Warmia and Mazury Province and tourism (i) is assumed to be a given branch of the economy, then the individual symbols should be understood as: $E_{i_{n}}$—employment in tourism in n-th municipality, $E_{T_{n}}$—total employment in n-th municipality, $\overset{N}{\underset{n=1}{\sum }}E_{i_{n}}$—employment in tourism in the entire province, $\overset{N}{\underset{n=1}{\sum }}E_{T_{n}}$—total employment in a given province. According to the above approach, the measure expressed by Formula (1) can be used to compare relative concentrations of employment in tourism of two municipalities.

The main disadvantage of the location quotient is the limited amount of economic information it conveys. It is designated only for one economic base and does not match modern wikinomics business models, such as digital prosumption and participation platforms. Nowadays, a new, synthetic measure of economic activity is needed, which would be appropriate for the digital economy. Such a measure should be based on teleinformatics and it needs to consider the regional diversity of municipalities, their mutual cooperation and it ought to allow the identification of regional poles and turnpikes of economic growth. It is also advisable that it not be limited to one branch of industry but includes all branches if possible. It is also necessary to consider municipal public administration offices, which—as the smallest units of the local government—are the initiators of economic activity in their subordinate areas. In this article we propose a measure that brings together all these key issues into one number.

## 3. The Four-Colour Theorem

Usually, the four-colour theorem is presented as follows: regions on each map drawn on the plane can be marked with only four colours in such a way that each two adjacent regions have different colours [20,21,22]. Adjacency signifies that the areas are bordering each other along a line. Cases of bordering in one point or even at a finite number of points are omitted. Region is understood as a fragment of the plane that is connected, and therefore consisting of only one area.

For normal maps, there is an upper limit on the number of neighbours that regions can border with. As Alfred Bray Kempe noted, any normal map plotted on a plane meets the following equation [23]:

(4) $$4p_{2}+3p_{3}+2p_{4}+p_{5}-p_{7}-2p_{8}-3p_{9}-\cdots -\left(N-6\right)p_{N}=12,$$

where $p_{n}$ represents the number of regions on the map that have exactly n neighbours, and N represents the largest number of neighbours any region can have. The formula starts with $p_{2}$, because cases when n=0 *i* n=1 are omitted. Normal maps do not have enclaves or islands. Each $p_{n}$ is either zero or positive, which is true for n<6. Equation (4) demonstrates that, at least one of the numbers $p_{2}$, $p_{3}$, $p_{4}$ or $p_{5}$ has to be positive, so that the left side of the equation is positive and corresponds to the right side. Therefore, one of the regions must have either two or three or four or five neighbours. There cannot be a normal map on a plane where each region would have six or more neighbours.

Each non-normal map can be assigned a normal map that can be drawn with at least the same number of colours. The truthfulness of the four-colour theorem for normal maps entails its truthfulness for all maps. The map of the United States is a good example as it is not a normal map because it contains the quadripoint, i.e., a single point at which the boundaries of the four states meet: Colorado, Utah, Arizona, and New Mexico. Yet, four colours are enough to colour it [21] (p. 5).

Two concepts appear in the context of the four-colour theorem: the unavoidable set and the reducible configurations. A set is called unavoidable if it contains a configuration set consisting of a region with two neighbours, a region with three neighbours, a region with four neighbours, or a region with five neighbours. The name of the unavoidable set results from the fact that each normal map must contain at least one of the four configurations listed. The reducible configuration occurs when it cannot appear on the minimal five-chromatic normal map, and thus the smallest normal map requiring the use of five colours. It is enough to find the unavoidable set of reducible configurations to prove the four-colour theorem.

The problem of four colours was formulated in 1852 by Francis Guthrie, who was then a student of mathematics at University College in London. During the colouring of the map of England, he noticed that four colours are sufficient to meet the condition that each two neighbouring counties have different colours. However, proving the four-color theorem turned out to be very difficult and, despite many attempts, it took mathematicians 125 years. The correct proof was announced in 1976 by Kenneth Appel and Wolfgang Haken, who based it on the construction of the unavoidable set of 1936 reducible configurations. Later it occurred that the number of these configurations was higher and finally amounted to 1482 [24]. The most interesting fact was that the proof was obtained on the basis of computer calculations. In the following years the proof was improved, but always required the use of a computer [25,26,27,28,29]. Validation of this proof requires a new type of reasoning, quite different from the one previously used in mathematics, which, perhaps, goes beyond the capabilities of the human mind. It sparked a discussion about the nature of a mathematical proof [30]. This problem even initiated some philosophical discussions around the four-colour theorem, which indicated that it was not an a priori truth, as required by classical mathematical tradition, but an a posteriori truth [31,32,33]. The map colouring problem seems to be a task worth a quantum computer [34].

In recent years, simpler proofs of the four-colour theorem have emerged, as exemplified by the proposals of the Polish mathematician Antoni Smoluk or John W. Oller, Jr. [35,36], but—so far—these works have not been the subject of wider scientific discussion. It has even been suggested that some of these proofs do not require the use of a computer as they can be fully verified by human mathematicians [37]. It is worth noting in this context that the complexity of the four-colour theorem corresponds to that of the Riemann hypothesis and is almost four times greater than the complexity of Fermat’s last theorem [38].

## 4. The Dual Graph

In this part, we will discuss the basic concepts of graph theory that will be needed in further research. A planar graph is understood as such a graph that can be drawn on the plane in such a way that no two edges intersect geometrically, except for the vertex, where they combine. As such a graph is represented on a plane, it is often called a plane graph. A connected graph is a graph in one piece, so any two vertices are connected by a path. For a planar graph G located on a plane, another graph $G^{*}$, called the (geometric) dual of G can be specified. Its construction takes place in two stages [39,40]:

(1) inside each face F of graph G we choose the point $V^{*}$; all these points will create the vertices of the new $G^{*}$ graph;
(2) corresponding to each edge E of the graph G we draw the line $E^{*}$ which intersects only the edge E (and no other edge of the graph G) and connects the vertices $V^{*}$ located on the faces F adjoining to E; these lines form the edges of the dual graph $G^{*}$.

The concept of duality has been known for a long time, it was noticed in antiquity that the dual of a cube is an octahedron, and that the dual of a dodecahedron is an icosahedron. The fact that the graph G is both plane and connected demonstrates, that a dual graph $G^{*}$ has the same properties. Moreover if a plane connected graph G has N vertices, E edges and F faces, and its geometric dual $G^{*}$ has $N^{*}$ vertices, $E^{*}$ edges and $F^{*}$ faces, then between the numbers of vertices, edges and faces of G and $G^{*}$ we observe the following relations: $N^{*}=F$, $E^{*}=E$, and $F^{*}=N$.

**Figure 1:**
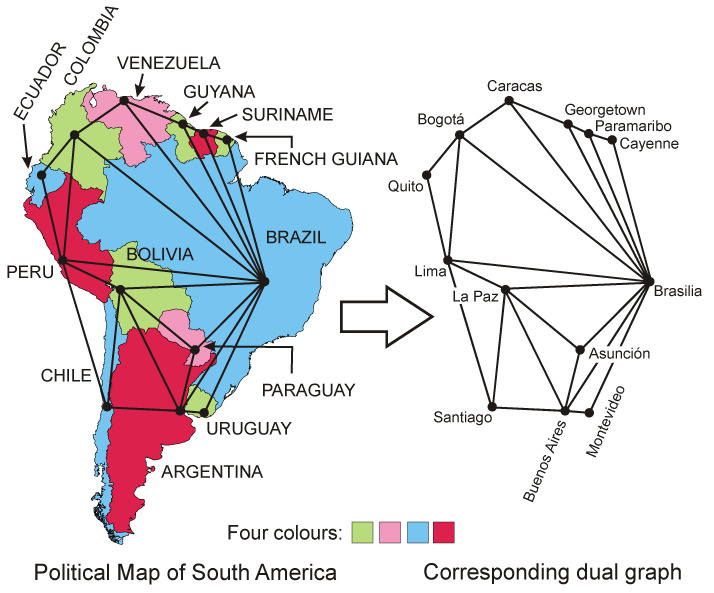
The political map of South America and the corresponding dual graph.

A map on a plane is a type of planar graph, so such a map also has a corresponding dual graph. The political map of South America was selected in order to present the idea of constructing a dual graph. The map was coloured according to the conditions of the four-colour theorem. The map includes 13 countries, within which there are 13 capitals. This map is a type of planar graph, in which the faces represent individual countries, the edges are inter-state borders, and the vertices form the points where the borders meet. If we connect the capitals of neighbouring countries with roads passing through their common borders, then we receive a dual graph of the original map. Capitals of the countries denote the vertices of the dual graph, the roads connecting the capitals of neighbouring countries are its edges, and the triangles resulting from the connection of capitals are faces. Figure 1 presents both the political map of South America and the corresponding dual graph. It should be emphasised that the edges of the dual graph are not real routes connecting national capitals, but conventional connections in the form of straight line segments.

## 5. The Model Graph Being the Reference System for Research

In spite of appearances the four-colour theorem does not have significant applications in cartography and geography. In fact, it can be stated that its role in science is twofold. First of all, it is an extremely important and unique mathematical achievement, especially with regard to the proof of the theorem, which initiated a philosophical discussion concerning cognitive capabilities of the human mind. So far, there is no chance that a human being could personally check the reducibility of all of the configurations in the unavoidable set. Secondly, from the point of view of other sciences, this theorem is still only a mathematical curiosity, because non-mathematical sciences lack its significant applications, i.e., those that would solve a major problem. So far, only a few attempts have been made to apply this theorem in various fields of science, but these endeavours have not led to significant progress [41,42,43,44,45,46]. We decided to break this deadlock. In 2018, we were able to prove that the four-colour theorem can be a useful tool for identifying spatial regional poles and turnpikes of economic growth [47]. Presently, we wish to proceed a step further and present that the theorem can be used to develop tools for measuring regional growth in the digital economy.

Dual graphs play a major role in the proposed method for identifying spatial regional poles and turnpikes of economic growth. The conversion of the map into a dual graph allows the study of a very significant economic problem, which is the cooperation of municipalities. Wikinomics participation platforms, i.e., websites of municipal public administration offices, are the basis of cooperation. On one hand, these websites allow municipalities, as the basic units of the local government, to support entrepreneurship in their subordinate areas, while on the other they enable the manifestation of creativity and innovation of the inhabitants of these municipalities in the form of digital prosumption. That is why the quality of websites plays a key role in the modern digital economy.

Two reference systems are used to assess economic growth in the Warmia and Mazury Province. The first is the dual graph, called the model graph, which corresponds to the map coloured according to the conditions of the four-colour theorem and is synonymous with a complete lack of growth poles. The second occurs when the websites of all municipalities belong to the highest quality class. In such a case, the dual graph is reduced to one vertex, and the whole province becomes one big growth pole and dominates the national economy. These two reference systems, although they have little chance of occurring in the real world, form a natural framework defining the scope of research. Everything that occurs in reality must be between these two cases. However, they point to the essence of the presented method used to identify spatial regional poles and turnpikes of economic growth. The method involves the reduction in dual graphs corresponding to real maps and comparing them with the dual graph corresponding to the reference map. Municipalities on real maps merge into clusters, which denote the quality classes of websites run by these basic local government units.

**Figure 2:**
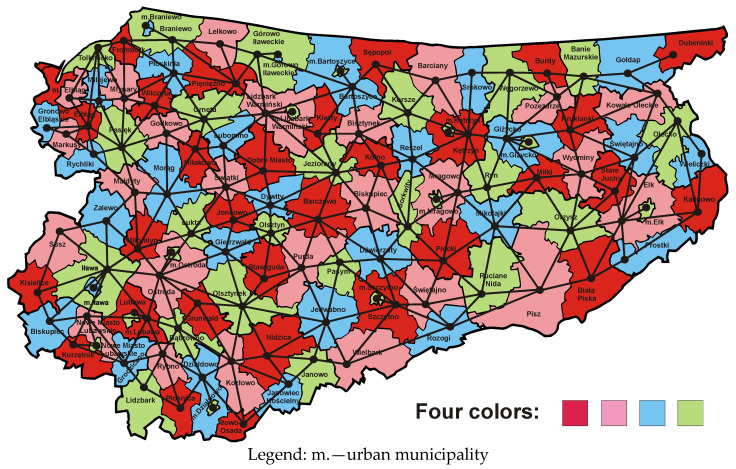
Map of the municipalities of the Warmia and Mazury Province coloured in accordance with the four-colour theorem and a corresponding dual graph. It is a frame of reference without growth poles.

According to these guidelines, at the beginning the map of the province was coloured in such a way that each two neighbouring municipalities were marked with different colours. This allowed the plotting of the corresponding dual graph. Subsequently, the capitals of all municipalities were marked on the map of the province, and then the capitals of adjacent municipalities were connected by roads passing through their common borders. Figure 2 presents the map of the province coloured according to the conditions of the theorem and the corresponding dual graph. The capitals of municipalities form the vertices of this graph, while the roads are its edges. The dual graph is plotted in such a way that all its edges are sections of straight lines. These edges divide the plane into areas called faces. The original map—after eliminating cases of complete surrounding of one municipality by another municipality—is a normal map on which each vertex joins precisely three edges. All dual graph faces are triangles because the faces are connected to the vertices of the original map. Consequently, such a dual graph is called triangulation.

In the dual graph, the number of edges ending in a given vertex is called the degree of the vertex and is equal to the number of country borders that on the original map correspond to that vertex. The circuit of the graph is called a path of edges, which has a beginning and an end in the same vertex, does not cross itself and divides the graph into two parts: its interior and its exterior. In the vocabulary of dual graphs, a configuration is understood as a part of triangulation, which consists of a set of vertices and all edges connecting them. The boundary circuit is called the ring of the configuration [48] (p. 166). Figure 2 shows that on the original map of the Warmia and Mazury Province there are thirty-nine external municipalities surrounding its internal part. After plotting the dual graph, they were replaced with the ring of the configuration, i.e., the boundary circuit, consisting of thirty-nine vertices and thirty-nine edges. The vertices of first degree, such as the municipality of Dubeninki, were omitted for obvious reasons. The configuration presented in Figure 2 in dual form is called the thirty-nine ring configuration, since its ring has thirty-nine vertices. It corresponds to the ring of thirty-nine municipalities that circle the original configuration.

## 6. Evaluation of Websites of Municipal Public Administration Offices

The main point of research was the assessment of websites of municipal public administration offices in the Warmia and Mazury Province in terms of their role as wikinomics participation platforms. The assessment consisted of sixteen points, which are presented in Table 1 [49,50]. The points were coded with symbols from A01 to A16. In order to obtain information about the quality of websites, a binary method was used, which consisted in assigning the value one to the website when the given criterion was met, or zero otherwise. Afterwards, all values assigned to individual websites were added together. Thus, the webpage functionality index included a number from the closed interval from 0 to 16.

The next stage of research involved the use of the k-means algorithm to divide websites into homogeneous subsets representing their quality classes [51]. The term cluster analysis was first used in 1939 by Robert Tryon, who was a pioneer in the study of various data classification algorithms [52]. James B. MacQueen started fundamental studies on the *k*-means clustering method [53], although the idea itself came from the Polish–Jewish mathematician Hugo Steinhaus [54], who dealt with this problem in 1956. In the 1970s, John A. Hartigan and M.A. Wong made a significant contribution to the improvement of the k-means clustering method [55,56], by proposing a suitable algorithm, finding recognition even today [57]. The well-known standard algorithm was developed by Stuart P. Lloyd in 1957, but the article on this topic was not published in the scientific journal until 1982 [58]. The problem consists of the division of the data set $X=\left(x_{1},x_{2},\cdots ,x_{n}\right)$ into a predetermined number of k clusters of greatest possible distinction. As a result of calculations, we obtain a set of k cluster centroids and an assignment of each point X to one cluster in such a way that the distances of all points belonging to a given cluster from the corresponding centroid are smaller than their distances from any other centroid. From the mathematical perspective, it is presented as follows:

(5) $$\arg\min\overset{k}{\underset{i=1}{\sum }}\underset{x_{n}\in C_{k}}{\sum }\|x_{n}-\mu_{k}\|{}^{2},$$

where $C_{k}$ and $\mu_{k}$ denote clusters and centroids, respectively. The division of the set of observations into k≤n cluster is based upon the minimisation of the within-cluster sum of squares, and thus variance.

It should be noted here that k-means clustering belongs to NP-hard problems [59,60,61,62,63]. In computational complexity theory NP (non-deterministic, polynomial time) it is the complexity class containing the set of decision problems that can be solved by a non-deterministic Turing machine in polynomial time [64] (p. 56). Most of the difficulties arising from the fact that *k*-means clustering belongs to the class NP-hard problems can be overcome by using an iterative method known as Lloyd’s algorithm. It consists of an alternate performance of two operations: (1) when a set of centroids $\mu_{k}$ is determined, the clusters $C_{k}$ are actualised by reducing—inside each cluster—the distance of points from the centroid; (2) based on the set of clusters, centroids which are the means of all points belonging to individual clusters are recalculated. These operations take the following form:

(6) $$C_{k}=\left\{x_{n}:\|x_{n}-\mu_{k}\|\leq \mathrm{all}\;\|x_{n}-\mu_{l}\|\right\},$$

(7) $$\mu_{k}=\frac{1}{C_{k}}\underset{x_{n}\in C_{k}}{\sum }x_{n}.$$

This procedure is continued until the assignments of clusters and centroids no longer change. The algorithm shows the convergence in few steps, but the solution can be in the form of a local minimum. In the worst case, Lloyd’s algorithm needs $i=2^{\Omega \left(\sqrt{n}\right)}$ iterations, and thus its running time is superpolynomial [65]. The used big-Ω notation regards the asymptotic lower bounds, and thus the limit of the growth of the running time of the algorithms from below for large enough input sizes [66]. For the Lloyd algorithm, the time complexity varies from Ω(n) to $2^{\Omega \left(\sqrt{n}\right)}$.

Table 2 presents the results of using the Lloyd’s algorithm to divide the websites of municipal public administration offices into wikinomics quality classes. Four clusters representing websites of low quality, average quality, high quality and very high quality were naturally separated. On the basis of this division, it is possible to determine not only empirical dual graphs, the edges of which represent barriers to inter-municipal cooperation, but also complementary graphs, the vertices and edges of which define, respectively, digital growth poles and development axes [67]. The study was conducted four times, i.e., in 2009, 2012, 2015 and 2019 [47,50]. Table 2 shows that most municipalities have high and very high quality websites, which demonstrates that these websites should be treated as the seeds of wikinomics platforms for participation. After some transformations these websites could perform the role of platforms for grassroots action, which would include platforms for public disclosure and platforms for neighbourhood knowledge. In this way, information held by public administrations would contribute to the creation of new values and services that would benefit both residents and entrepreneurs. Such actions would certainly lead to the development of entrepreneurship and an increase in the regional economic growth rate and the level of prosperity. In addition, the high quality of websites would contribute to the development of digital prosumption, which would enable the use of the natural innovation and creativity of people. In the processes of prosumption, the difference between the producer and the consumer, the service provider and the recipient disappears, which allows the passive party, the consumer or the recipient, to participate in the design, creation and production of goods or services. However, new business models in the form of prosumption and participation platforms can only function well if the four basic principles of wikinomics are met: openness, peering, sharing, and acting globally. Under these conditions, it is necessary to give prosumer communities some control over the product or service. In this case, it involves co-creating some public administration services by the local government, residents and entrepreneurs.

## 7. Determination of Growth Poles and Development Axes According to the Dual Graph Reduction Method

Empirical maps and the resulting dual graphs were created in a similar way to the reference map and the reference dual graph. The only difference is that each municipality received a colour corresponding to the quality class of its website run by the administration office operating in its area. After the map of the examined province was coloured in accordance with the quality classes of websites, it occurred that some neighbouring municipalities have the same colours. If these colours correspond to classes of high or very high quality, then it is assumed that the given municipalities form spatial poles or even regional axes of growth and development when there are more municipalities. Generally speaking, the same colour of neighbouring municipalities, i.e., having websites of the same quality, is interpreted as a form of cooperation between the municipalities. The empirical dual graphs of the studied province were created on the assumption that a set of neighbouring municipalities, marked with the same colour, is treated as one region. In this case, the contractual capitals of all regions or even individual municipalities are marked with points, provided that they are separated from the surrounding, and then connected with sections of straight lines passing through the common borders of these regions or municipalities. In this way a dual graph corresponding to the real map is created. It is reduced in comparison to the model dual graph corresponding to the map coloured according to the four-colour theorem. The reduction in the edges of empirical dual graphs indicates inter-municipal economic cooperation. However, the existence of edges connecting neighbouring municipalities indicates the existence of certain growth barriers, because the websites belong to different quality classes.

Enterprises and sectors of the economy which initiate economic activity in a given area and contribute to better economic results of enterprises and industries operating in their environment are propulsion units that form regional poles of growth. The growth pole theory was developed in the 1950s by the French economist François Perroux [68], who included in his research many innovative elements that are the basis of today’s complexity economics [69]. According to wikinomics, a characteristic feature of a growth pole is the initiation of economic activity with use of advanced ICT tools. A website of the municipal office of public administration, which acts as a participation platform and integrates and strengthens the economic forces operating in the territory of a given municipality can be such a pole. The higher the quality of a website, the greater the chance that it will become a local growth pole.

The theory of development axes elaborated in 1963 by Pierre Pottier is closely connected with the theory of growth poles, according to which economic development is spreading along trade routes and transport networks connecting major industrial centres [70,71]. Territories located between growth poles and providing transport communication receive additional growth impulses due to the increased flow of goods, the spread of innovation and the development of infrastructure. Therefore, they transform into development axes (corridors), which together with the growth poles define the spatial framework for economic growth of a large region or country. The axis concept helps to connect the transport network with the urban hierarchy theories and growth centres into a single unit. These concepts can be easily adapted to the research regarding the Warmia and Mazury Province. Currently, the role of the development axes is played not only by transport networks, but also by computer networks. In the digital economy, the role of ICT is at least as important as traditional trade routes. The spatial distribution of municipalities that have high or very high quality websites form wikinomics poles of growth, indicates through which paths the economic growth spreads in space and where barriers to growth appear. In this way, digital growth turnpikes or development axes can be identified in the economic space. The lack of edges between neighbouring municipalities means that the municipalities have websites belonging to the same quality class. The high or very high quality of these websites means that development axes are created between these municipalities. However, if there are edges between neighbouring municipalities, it can be said that there are growth barriers between them.

**Figure 3:**
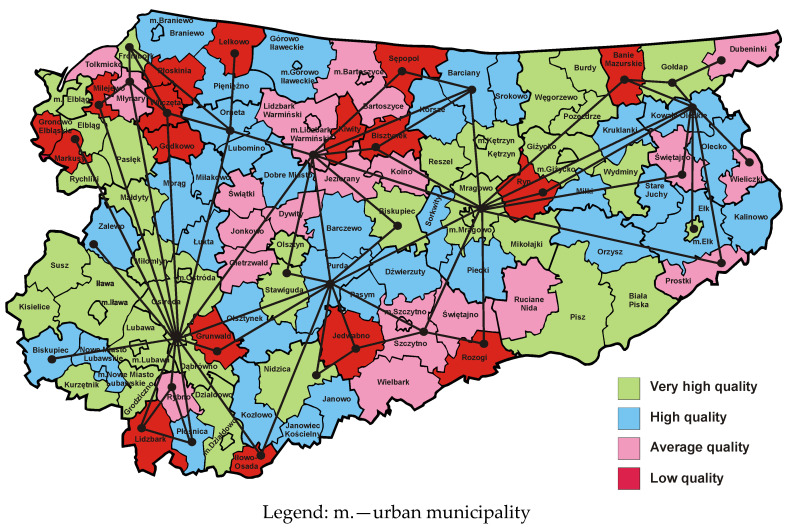
The map of the municipalities of the Warmia and Mazury Province divided into categories of the quality of the websites maintained by municipal public administration offices in 2009 and a corresponding dual graph.

Figure 3 presents a map of the Warmia and Mazury Province, where municipalities are marked with colours representing the quality classes of websites maintained by municipal public administration offices in 2009 [50]. A comparison of dual graphs corresponding to the reference map based on the four-colour theorem and the empirical map from 2009 indicates that the impact of local growth poles on the area is associated with a reduction in the number of vertices and the number of edges of the graph formed on the basis of the actual map. The dual graph shown in Figure 3 has significantly fewer vertices and edges than the dual graph associated with the reference map with no growth poles. In this way essential information regarding regional economic growth is presented. The dual graph associated with the empirical map from 2009 indicates a particular feature of the province in question. Overall, it is dominated by historically and politically conditioned infrastructural growth poles, that are closely related to road, rail and inland waterway infrastructure existing in the Warmia and Mazury Province [47]. Historical considerations result from the fact that this infrastructure is the effect of the work of many previous generations that have built it over the last several hundred years.

The infrastructure currently existing in the studied province is also the result of political history, which dates back to at least the 13th century. In the years 1226–1466 the area was ruled by the Teutonic Order (Monastic State of the Teutonic Knights). The order contributed to the economic development of these lands by introducing technical progress in the form wind milling improving the process of grinding and replacing the previously used hand-mills [72]. As a result of the Second Peace of Thorn, which was signed in 1466, the State of the Teutonic Order was split into Royal Prussia and Teutonic Prussia (since 1525 Ducal Prussia). Both parts of Prussia were actually attached to Poland, but the latter was dependent on fiefs [73,74]. The period from the 15th to the 18th century (and also a little later) was a golden period of development of windmills in Warmia and Mazury. At that time, these areas were formally or informally part of the Polish economy. The location of windmills in this area was consistent with records from the turn of the 19th and 20th centuries, and closely correlated with the existing transport infrastructure. Thus, windmills have marked the basic, existing to this day, trade and communication routes. To conclude these historical remarks, it should be noted that a significant part of the Warmia and Mazury Province later belonged to East Prussia and it was only in 1945 that it was incorporated into Poland as part of the Recovered Territories. The studied province currently borders on the north with the Kaliningrad Region belonging to Russia. To sum up, the infrastructure currently existing in this area is not only the result of a specific economic history, but also of political history.

As noted above, high and very high quality municipal websites are closely linked to the transport infrastructure created in previous years. A cluster of municipalities with very high quality websites is in the western part of the province and it creates a development axis located on the north–south line. The transport infrastructure in this area comprises of four elements:

(1) the Ostróda–Elbląg Canal, which was built in the 19th century;
(2) Elbląg port, serving cargo barges floating on the Vistula Lagoon, improving trade exchange between Poland and Russia;
(3) road connections Nidzica–Olsztynek–Ostróda–Pasłęk–Elbląg–Braniewo–Królewiec with two road border crossings Gronowo–Mamonowo and Grzechotki–Mamonowo II;
(4) Malbork–Elbląg–Bogaczewo–Braniewo–Królewiec railway line with the Braniewo–Mamonowo railway border crossing.

The second cluster of municipalities with the highest quality of websites is located in the eastern part of the province and creates a development axis also running along the north–south line. There are two national roads in that part of the province. The first of them (No. 63) runs through Pisz, Orzysz, Giżycko, Węgorzewo and before 1945 led to today’s Kryłów in the Kaliningrad Region (on the Russian side). Currently there is no border crossing there. The second road (No. 65) runs through the cities of Ełk, Olecko, Gołdap and ends with the Polish–Russian road border crossing Gołdap–Gusiew. On the map presented in Figure 3 it is possible to notice the third cluster in the form of a strip of municipalities with high and very high quality sites located in the central part of the province, which is located on the southwest–northeast line. The national road No. 16, which connects towns such as Iława, Ostróda, Olsztyn, Mrągowo, Orzysz and Ełk, and then crosses the Podlasie Province and runs up to the border with Lithuania in Ogrodniki forms the main communication route in this region. This cluster has a railway line No. 353 connecting the towns of Iława–Ostróda–Olsztyn–Czerwonka–Korsze, which ends with the Skandawa–Żeleznodorożnyj railway border crossing. The fourth cluster, slightly less visible, is connected with the national road No. 51, which connects the towns of Olsztynek, Olsztyn, Dobre Miasto, Lidzbark Warmiński, Bartoszyce and ends with the Bezledy–Bagrationowsk border crossing. To sum up, the empirical map from 2009 together with the corresponding dual graph (Figure 3) allows the location of four clusters of municipalities with regional growth poles connected by a transport network. The Warmia and Mazury Province is therefore one of the best examples of the correctness of the development axes concept proposed by Pierre Pottier. It is also worth noting that the barriers to growth and development, represented by the edges of the dual graph, are located near the northern and southern borders of the province.

**Figure 4:**
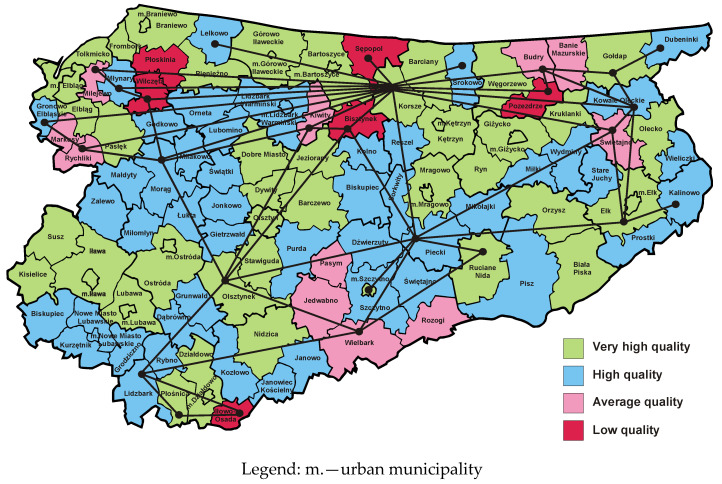
The map of the municipalities of the Warmia and Mazury Province divided into categories of the quality of the websites maintained by municipal public administration offices in 2012 and a corresponding dual graph.

Figure 4 presents a map of the Warmia and Mazury Province, in which municipalities are marked with colours representing the quality classes of websites operated by municipal public administration offices in 2012 [50]. It can be observed that in comparison to 2009, the number of municipalities with high and very high quality websites has increased significantly. The dual graph from 2012 has significantly fewer edges than the dual graph from 2009. This demonstrates the emergence of new growth poles and development axes that have contributed to the elimination of many barriers to inter-municipal cooperation. Barriers located near the northern border of the province could not be removed, so investment and innovation should be continued in this area.

The investment in the construction of fibre-optic backbone distribution network with a length of 2292 km along with the appropriate infrastructure significantly contributed to the rapid progress in the development of municipal websites in 2012 [75,76]. The network consists of 216 distribution nodes, 10 backbone nodes, 2 contact points to a higher-level fibre-optic network and two network management centres. In the distribution layer, the network provides access with a bandwidth of at least 30 Mbit/s, and in the backbone layer at least 100 Gbit/s, thus meeting the requirements of next generation access (NGA) networks. The purpose of this project was to provide access to broadband internet to residents, enterprises and government institutions in the Warmia and Mazury Province. This investment was carried out in the years 2008–2015 and its value amounted to PLN 327,041,042.07 (approx. EUR 71,559,490.65). It was financed by the European Regional Development Fund, the state budget and the province’s own contribution. The project was implemented on the basis of public–private partnerships as part of the design, build, operate, transfer (DBOT) model. The Warmia and Mazury Province is the owner of the created infrastructure, while the private partner was obliged to design, build, manage and operate the telecommunications infrastructure until the end of 2025. After this time, all generated assets will be transferred to the public partner.

Investment in the development of telecommunications infrastructure is crucial for the region’s economy, as it will accelerate its development and facilitate the introduction of innovative technologies. Computer networks form the basis of digital economy, contribute to the intensification of modernisation processes of existing enterprises and stimulate the development of new products and services provided with use of the Internet. This type of growth refers to the well-known economic turnpike theory [77,78]. This name comes from an American English word meaning highway. Let us assume that we want to put the economy on the sustainable growth path, which is the maximum rate of growth in the sense of von Neumann. This path is associated with the top technique, which gives the highest rate of return. The solution resembles a dilemma of a driver who wants to reach his destination as soon as possible and has a choice between using the turnpike and the minor roads. Most often, the best choice turns out to be the use of a turnpike, even if it involves incurring costs at intermediate stages. This development method was chosen in the studied province, because according to wikinomics, the information and communication technique is now the top technology. Thus turnpikes can appear both in economic spaces, described by mathematical growth models, and in physical spaces, creating development axes defined by Pierre Pottier.

**Figure 5:**
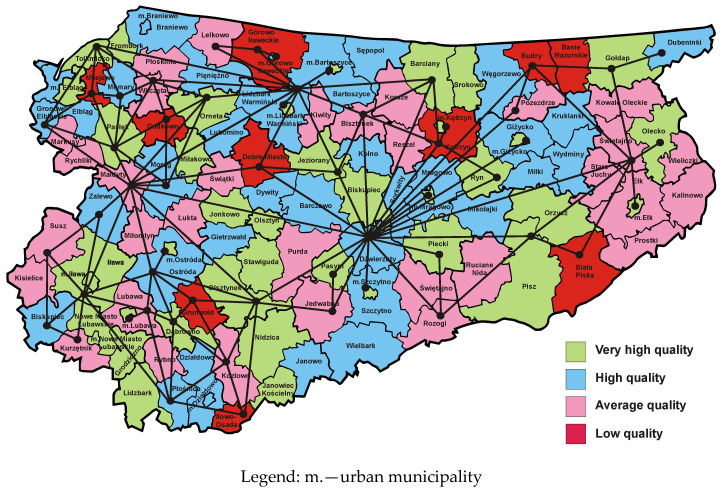
The map of the municipalities of the Warmia and Mazury Province divided into categories of the quality of the websites maintained by municipal public administration offices in 2015 and a corresponding dual graph.

Figure 5 presents a map of the Warmia and Mazury Province, in which municipalities are marked with colours representing the quality classes of websites maintained by municipal public administration offices in 2015. It can be observed that the number of municipalities with high and very high website classes has heavily decreased. Thus, the number of municipalities with websites belonging to low or medium quality class has increased. At the same time, in comparison to 2012, the number of vertices and dual graph edges associated with the empirical map of 2015 increased, which can be interpreted as the emergence of new development barriers hampering the region’s economic growth. The reasons for this state of affairs should be sought in the sphere of economic policy. The deterioration in the quality of websites may result from the fact that on 15 September 2015 all construction works related to the broadband network discussed above were completed. The network is backbone-distribution; therefore, its implementation does not signify an immediate improvement in internet access for residents, institutions and enterprises. It is also necessary to build the last mile networks, i.e., access networks, which will connect individual end users to the main network. This task was entrusted to local telecommunications operators, who on an equal basis can provide internet access services to interested entities from the Warmia and Mazury Province.

The decrease in the quality of many websites of municipal public administration offices can be explained by the fact that delays related with the construction of the last mile network were not taken into account and existing internet connections, such as radio networks, were too lightly abandoned. To prevent delays, the Digital Plan 2025 for Warmia and Mazury was implemented, which aims to eliminate or reduce economic barriers that would hinder the construction of the last mile networks in the studied region [79,80]. This plan is a unique agreement in nationwide scale signed between the Province Board and the authorities of districts and municipalities from the region. As part of it, fees for placing telecommunications infrastructure along public roads have been reduced and unified, and this infrastructure has been exempted from property tax, in order to reduce the price of internet access for the end user. This programme is treated as a flywheel for the socio–economic development of the region, as it is intended to support the development of an innovative, low-carbon economy and direct entrepreneurship to services and products offered through a global network. It is worth noting that the internet network in the Warmia and Mazury Province is strongly correlated with the locations of windmills in the past centuries, most of which are gone today. As mentioned, windmills had a decisive impact on the formation of the transport network in the region. Over time, old trade and merchant routes were transformed into today’s public roads. The development of modern road infrastructure was based on historical trade routes, which proved to be an economically advantageous solution. This is due to the geographical diversity of the land surface in Warmia and Mazury, where there are many rivers, streams, forests and thousands of lakes. Construction of new roads overcoming these obstacles would be too expensive, although such projects are undertaken in special cases, such as bypasses of major cities. The fact that the telecommunications infrastructure runs along ancient roads shows that the development axes in the region under review are extremely durable. In other words, not only growth poles, but also development axes are the result of historical and political considerations.

**Figure 6:**
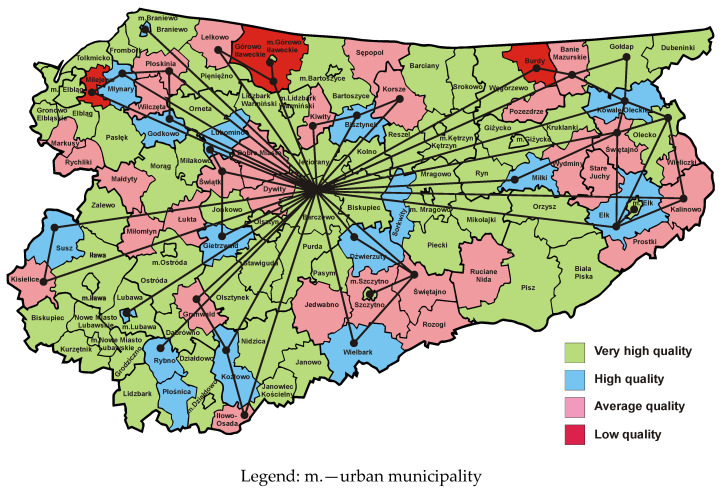
The map of the municipalities of the Warmia and Mazury Province divided into categories of the quality of the websites maintained by municipal public administration offices in 2019 and a corresponding dual graph.

Figure 6 presents a map of the Warmia and Mazury Province, in which municipalities are marked with colours representing the quality classes of websites maintained by municipal public administration offices in 2019. As is easy to see, the number of municipalities with websites included in the classes of high and very high quality has significantly increased. Some barriers that prevented inter-municipal cooperation in previous years have also disappeared. This signifies that new digital growth poles and development axes have emerged in the surveyed province. These changes prove that the Digital Plan 2025 for Warmia and Mazury is being implemented gradually. Nevertheless, the empirical dual graph from 2019 demonstrates that there are still many barriers to development, especially in the northern and eastern parts of the region.

## 8. Descartes’ Theorem, Euler Characteristic, and Cauchy Modification

The determination of the measure of economic growth of the Warmia and Mazury Province requires designating the relationship between the numbers of vertices, edges and faces in the dual graph. René Descartes (1596–1650) was the first to study the general properties of polyhedra. He presented the basic theorem regarding the problem in the work *Progymnasmata de Solidorum Elementis* (Exercises on the Elements of Solids) [81]. It reads as follows:

**Theorem** **1.** *The sum of the deficiencies of the solid angles in a polyhedron is always eight right angles.*

The deficiency of a solid angle is the amount by which the sum of its face angles is smaller than 360°. The size of deficiency determines the sharpness of the solid angle in such a way that the greater the deficiency the more acute the angle. In a cube, the deficiency of each solid angle is 90°, whereas in a dodecahedron the deficiency of each solid angle is 36°. It is worth noting that in both cases the deficiencies of all solid angles amount to 720°, which equals eight right angles (the whole sphere) [82] (pp. 187–189). Based on this theorem, Descartes formulated two statements:

1. There are always twice as many plane angles as sides [*E*] on the surface of a solid body, for one side is always common to two faces.
2. I always take α [*V*] for the number of solid angles and ϕ [*F*] for the number of faces. The actual number of plane angles is 2F+2V−4.

Symbols V, E, and F in square brackets are abbreviations of modern equivalents of terms used by Descartes: numbers of vertices, edges, and faces of a polyhedron accordingly. The following relationship results from the above:

(8) Number of plane angles= 2F+2V−4=2E,

which gives the basic result:

(9) F+V−E=2.

Perhaps Descartes was unaware of the existence of such a relationship in a polyhedron [83] (p. 515), but it is not certain. There is no proof of this theorem in his work. However, Descartes did not publish the work *Progymnasmata*, its content survived as a result of several unusual events. The history of this manuscript is described by Peter Cromwell in his book *Polyhedra* [82] (pp. 181–182):

*In the autumn of 1649, Descartes went to Stockholm at the invitation of Queen Christina of Sweden, but the severity of the climate was too much for him and he died six months later. His belongings were shipped back to France but suffered accident on route, the box carrying his manuscripts ending up in the Seine at Paris. The papers were rescued from the river, separated and dried. Later, some were published and the remainder were made available for consultation. In 1676 Gottfried Wilhelm Leibniz (one of the founders of the calculus) made copies of several of the latter manuscripts including the work on polyhedra. Descartes’ original manuscript has vanished and it is only through the copy that the work is preserved. Even so, it remained unknown until 1860 when the copy was discovered by Comte Foucher de Careil among a collection of uncatalogued Leibniz papers.*

Leonhard Euler (1707–1783)—Swiss mathematician and physicist—was another researcher who began studying the general properties of polyhedra. At the time mathematicians used to describe their findings in letters to friends. This was due to the long waiting time for publication of results. Euler corresponded for many years with the German mathematician Christian Goldbach (1690–1764). In a letter dated 14 November 1750 Euler wrote to Christian Goldbach that he had begun studying polyhedra. The Swiss scholar aimed to categorise the properties of various and seemingly unrelated solids. Below is an excerpt from his letter [84] (p. 76):

*Recently it occurred to me to determine the general properties of solids bounded by plane faces, because there is no doubt that general theorems should be found for them, just as for plane rectilinear figures, whose properties are:*

*(1)*  that in every plane figure the number of sides is equal to the number of angles, and
*(2)*  that the sum of all the angles is equal to twice as many right angles as there are sides, less four.

*Whereas for plane figures only sides and angles need to be considered, for the case of solids more parts must be taken into account*…

Euler wrote two papers on the polyhedral formula that were published in 1758. The first contains the statement of the theorem [85], while the second contains the poof [86]. The theorem given by Euler has the form:

**Theorem** **2.** *In every solid enclosed by plane faces, the number of faces along with the number of solid angles exceeds the number of edges by two.*

Using previously proposed symbols the relation can be presented as follows:

(10) F+V=E+2,

hence

(11) χ=V−E+F=2,

where χ stands for the Euler characteristic. The number χ is a topological invariant, as it describes a topological space’s shape or structure. Euler aimed to classify all polyhedra, by developing the theory of stereometry (solid geometry) in the image of existing planimetry (planar geometry). However, in the above theorem, he indicates that the considered polyhedra are enclosed by planes, therefore convex polyhedra, which is a sufficient condition for the truthfulness of his formula. Euler, however, was not able to provide a rigorous proof of the theorem [87,88,89].

In 1813, French mathematician Augustin-Louis Cauchy (1789–1857) generalised the Euler characteristic by projecting the polyhedron onto a plane. One of the faces of the polyhedron is assumed as the basis, and then all the other vertices are transferred to it without changing their number, which gives a set of polygons within this chosen face. In other words, the result of flattening of the polyhedron is a plane network of polygons. In this way Euler’s formula can be presented in the form of planar graphs or equivalently as maps on the plane. Cauchy’s theorem has the following form [90] (p. 77):

**Theorem** **3.** *If a polyhedron is decomposed into as many others as we choose, by taking at will new vertices in the interior, and if the number of new polyhedra so formed is denoted by P, the total number of vertices including those of the original polyhedron by S [V] the total number of faces by F, and the total number of edges by A [E], then*

(12) V+F=E+P+1,

*that is, the sum of the number of vertices and that of the faces exceeds by one unit the sum of the number of edges and that of the polyhedra.*

Euler’s theorem is a special case of Cauchy’s theorem. After assuming that all the polyhedra are reduced to a single one, which is equivalent to P=1, the Equation (10) is obtained. Another theorem that applies to plane geometry can also be derived from Cauchy’s theorem. Let all polyhedra be reduced to one. Let us assume that the last polyhedron will be transformed so that all the other vertices will be projected onto the selected face, without changing their number. This means substitution P=0 in Equation (12), which gives

(13) V+F=E+1.

This leads to the conclusion that the sum of the number of vertices and the number of faces exceeds by one unit the number of edges. Theorem (13) in plane geometry is the equivalent of the general theorem (12) in the geometry of polyhedra. Both theorems have been proved by Cauchy [84] (pp. 79–83).

## 9. Measurement of Economic Development in the Province of Warmia and Mazury

The proposed method of economic growth measurement is based on dual graphs, one of which—corresponding to the model map—plays the role of a reference system, while the others contain information about growth poles and development axes in individual years. The essence of economic growth measurement consists in comparing information contained in empirical graphs with the model graph. The latter is an absolute reference system, which makes our measure resistant to relative comparisons. It is something like absolute time in the sense of Isaac Newton. This situation in science is extremely rare, because all reality is constantly changing. The four-colour theorem gives us a kind of anchor that establishes dynamic and lasting links between an objective mathematical being—which is the four-colour theorem—and the changing economic situation. Usually in science we are constricted to relative comparisons. Calculation of real amounts of national income when we have nominal amounts of this income in individual years is a good example of this problem. The only way to solve this dilemma is to adopt the national income from a given year as the base value, and then determine the income in the remaining years at prices from the base year. This is an assumption that must be made and on which the calculation result depends. In the measure of economic growth presented here, this assumption is unnecessary.

The next step of the proposed method is the reduction of the dual graph. It happens when two bordering municipalities have web pages belonging to the same quality class. The removal of the edge means that the barrier of local growth has been overcome, which allows both municipalities to grow together which in turn may allow them to become the seed of a new growth pole. The edges of the dual graph correspond to the growth barriers, so they should be calculated precisely. For this purpose, the Euler characteristics χ(S) for convex polyhedra can be used as follows:

(14) χ(S)=V−E+F=2,

Due to the need for economical interpretation of the dual graph it is better to apply the Cauchy interpretation of the above formula, which is applicable in plane geometry:

(15) χ(G)=V−E+F=1.

In this way, we focus only on the dual graph itself, which is a representative of the contribution of the public administration sector to the economy of the Warmia and Mazury Province. In this case, we are not interested in the environment, and thus in the calculation of χ(G), the infinite (unbounded) face outside the dual graph, which contains a point in infinity, is not counted. Table 3 presents the results of calculating the number of vertices, faces and edges of all the dual graphs discussed above with the use of the Cauchy formula.

The definition of a new measure of the region’s economic development ($M_{D}$) based on the four-colour theorem is as follows:

(16) $$M_{D}^{Year}=1-\frac{\begin{array}{c} Sum\;of\;edges\;of\;the\;dual\;graph\;on\;the\;real\;map \end{array}}{Sum\;of\;edges\;of\;the\;dual\;graph\;on\;the\;reference\;map}\;.$$

The limit values of the fraction in the above formula are as follows:

0—(no edges) dual graph has only one vertex ⇒ all municipalities cooperate with each other ⇒ $M_{D}^{Year}=1.$

1—(all edges) no municipality cooperates with any other ⇒ empirical map = reference map ⇒ $M_{D}^{Year}=0.$

This measure, apart from being economic in nature and referring to the equipment of the public administration sector in the latest telecommunications technologies, also takes into account the spatial aspects of economic development. It can be applied to any region that consists of smaller and simultaneously autonomous spatial units.

The proposed measure in one number contains a complex set of information, and at the same time it is simple to use. It can therefore be used in interregional research, and it also allows intertemporal analyses. It has both a static and dynamic character. It can be used to identify spatial growth poles and development axes in the public administration sector, and thus also in the entire regional economy, in this case the province economy. However, it is required that there are public administration offices in the smallest surveyed units (municipalities) belonging to a higher-order unit (province).

The calculation method of spatial economic growth measures in individual years in accordance with the Formula (16) and the data contained in Table 3 is as follows:

(17) $$M_{D}^{2009}=1-\frac{63}{277}=0.7726,$$

(18) $$M_{D}^{2012}=1-\frac{49}{277}=0.8231,$$

(19) $$M_{D}^{2015}=1-\frac{106}{277}=0.6173,$$

(20) $$M_{D}^{2019}=1-\frac{58}{277}=0.7906.$$

The result can be rounded to four decimal places. Establishing certain thresholds defining the development phases of the region requires similar research to be carried out in all provinces, i.e., on the scale of the entire national economy.

Interpretation of the indicators points to a conclusion that that despite the increased financial outlays, public administration in the Warmia and Mazury Province is a weak factor of economic growth. Taking as a basis the assessment of the starting situation from 2009, we can see a great progress in 2012, intensified inter-municipal cooperation, as evidenced by the fact that many edges of the dual graph are reduced. New local growth poles appear and new ways of spreading economic growth are formed. However, in 2015 regress occurs and the situation becomes worse than in 2009. This should be connected with the completion of the construction of a fibre-optic network, which allowed access to broadband internet for businesses in almost the entire region. This means that public administration is a very sensitive sector for changes in financing. The source of this financing is mainly government spending. It is true that the network was built, but apparently it was forgotten that it is not a one-time investment and that it requires further government spending to properly use it. However, a remedy in the form of the Digital Plan 2025 for Warmia and Mazury was found relatively quickly [79]. The situation improved significantly in 2019, which is a sign of clear progress in the construction of the last mile networks.

There are interconnections between the growth poles and development axes in the form of the municipal digital platforms and the transport infrastructure. One is a mirror image of the other. Strictly speaking, there are feedbacks between them, which can be both positive and negative. Certainly, the construction of the fibre-optic backbone distribution network will have a great impact on the expansion of the transport infrastructure and thus contribute to the economic development of the region. However, infrastructural changes are slow, so the economic development measure expressed by the Equation (16) may be a leading indicator. According to the latest research, in Warmia and Mazury the synthetic index of regional innovation development has shown a steady decline since 2009. In 2009, this index was 0.27, in 2014 it was 0.24, and in 2019 it decreased to 0.22 [91]. After comparing it with the new measure of economic development introduced in this article, it is clear that digital growth poles and development axes precede investments in the expansion of transport networks, which form the main routes to prosperity and welfare in the region. As infrastructure-based economic growth is slow, the time gap between the two indicators can range from several months to even several years.

## 10. Discussion

The application of k-means clustering led to the creation of four quality classes for websites of municipal public administration offices in the four examined years. As presented below, this classification of websites is natural in a topological sense. Moreover, the diversification of the examined objects from a static point of view (one point in time, i.e., a specific year) and dynamic point of view (four points in time or more) allows the drawing of more far-reaching conclusions. Changes taking place in quality classes—represented by the transformation of dual graphs—prove that the Warmia and Mazury Province is a complex adaptive system. A complex adaptive system should be understood as an object that exchanges energy, matter and information with the environment; furthermore it functions based on cognitive schemas and has the ability to tune to the edge of chaos [92]. It is therefore an open system showing emergence. This conclusion can be justified on the basis of the catastrophe theory, which is a method of classifying stable forms [93,94,95,96,97,98,99,100,101].

Catastrophe theory is based on Thom’s classification theorem [102,103,104]. It indicates that any singularity of the catastrophe map is equivalent with one type of singularity belonging to a finite family of types, which are called elementary catastrophes. In addition, the catastrophe map is locally stable at all manifold points due to small perturbations. The number of elementary catastrophes depends only on the codimension (the number of control variables). Elementary catastrophes describe all possible ways of manifesting discontinuities in dynamical systems. Considering the examined problem of the division of websites into four quality classes, the case of codimension =3 is essential. Then the control space can be interpreted as physical space–time, which consists of two spatial dimensions and one time dimension. All events that occur in this space–time rely on the transformations of the dual graph on the plane, which occur over time. The catastrophe map then has five types of singularities. Four elementary catastrophes can be attributed to four quality classes of websites of municipal public administration offices, while the fifth catastrophe is related to the environment, and thus to what is located outside of the province under study. This interpretation of reality seems to be confirmed by life itself. The division of a three-dimensional entity, consisting of a two-dimensional object located on a plane and changing in time, into four quality classes is therefore natural. In other words, the compliance of the division of websites into four quality classes with Thom’s classification theorem proves that this division was made correctly. The fifth catastrophe, representing the space located outside of the examined province, emphasises the fact that the examined province is an open system that exchanges matter, energy and information with the environment, i.e., with the rest of the national economy or even the world economy. It is worth noting that monetary flows that enable the functioning of municipal public administration offices and initiate the economic development of the province originate from the space outside of the province.

The studied province is a complex adaptive system because it can be described by a measure called total information, which is the sum of effective complexity and an entropy term [105]. Effective complexity is the length of a compact description of the regularities identified in the examined system, and thus describes the object’s rule-based features. The effective complexity of the studied province is represented by dual graphs. Whereas the entropy term measures the information necessary to describe the random aspects of the entity. Entropy can also be understood as an ignorance measure based on Shannon informational entropy [106,107]. In this case, ignorance should be understood as the degree of unawareness of the national economic centre regarding the investment needs of the region.

One of the issues that need clarification is the impact of the geographical line complexity on results obtained through the four-colour theorem. Borders between countries, provinces and municipalities are not always smooth lines, sometimes they can have a very complex shape, depending on the terrain. The Warmia and Mazury Province is very diverse in terms of nature, has over 3000 lakes, many rivers and streams, and more than a third of its surface is covered by forests. The topography is mostly lowland, but hills and valleys occur in many places. In such conditions, it is often the case that the geographical lines separating individual municipalities are complex. It seems logical that the longer the common border between the two municipalities, the greater the likelihood that they will undertake economic cooperation and thus transform into one pole of growth. However, there may be exceptions to this rule when neighbouring municipalities compete for some common resources.

English researcher Lewis F. Richardson was the first to apply a topological approach to study armed conflicts between states [108,109]. He considered various causes of wars, and one of them was spatial relations. In particular, he studied the impact of common borders between the two countries on the emergence of conflicts. Richardson used the Euler characteristic for this purpose, which presents the relationship between the numbers of vertices, edges and faces in the polyhedron. It is established that during the period he studied, i.e., the years 1820–1950, there were about 60 stable nations and empires in the world. This allowed him to demonstrate that for any plausible arrangement of nations, the average number of neighbours for each of these countries should be around six. So, if countries seeking war would select their enemies entirely at random, there would be a ten percent chance that every pair at war would have a common border. Richardson’s research, however, showed that in the period under review, out of 94 international wars, which had only two participants, only 12 cases concerned combatants that had no common borders. This leads to the conclusion that close neighbourhood can be one of the main causes of conflicts.

Richardson found that the result of measuring the length of complex geographic lines, such as sea coasts or borders between countries, depends on the unit of measurement [110] (p. 26). The measured length is the greater the smaller the yardstick. This is because a shorter ruler measures more accurately the sinuosity of coastlines or land borders than a longer ruler because it takes greater account of the roughness. According to intuition, one would expect that taking smaller and smaller units of measurement will cause the total of the measurements to tend to a certain finite number representing the true length of the geographical line. However, it has been established to be otherwise. If the length of the meter decreases to zero, then the length of the shoreline or boundary tends to infinity. This phenomenon is now known as the Richardson effect. In this context, the coastline paradox is also discussed. It can be concluded that the length is not an adequate feature to describe a geographical line, because its length is simply undefinable. Geographical lines are actually fractals that can be described by the fractal dimension which is determined by the slope of a straight line obtained by plotting the length of the ruler versus the measured length of the geographical line on a log–log plot [111,112]. This is how Richardson discovered fractals [113] (p. 260).

There are similarities between the measure of economic development of a separate spatial object (province) based on the four-colour theorem proposed in this article and Richardson’s research on the causes of armed conflicts. Richardson focused on the study of spatial relations, which prompted him to use dual graphs to analyse the data collected on international wars. Modern reconstructions show that these graphs were very complicated [114]. War was marked on the map with an edge connecting the countries involved. In addition, the thickness of the edge reflected the magnitude of the conflict. Richardson classified wars and other quarrels on the basis of a method borrowed from astronomy, which involved the use of common logarithms, i.e., the base-10 logarithms. Hence a terror campaign, during which 1000 people die has a magnitude of 3 $\left(\log10^{3}=3\right)$, and a war with 10 million casualties has a magnitude of 7 $\left(\log10^{7}=7\right)$.

In Richardson’s research, the edges of dual graphs denote armed conflicts and their magnitudes, and thus—generally speaking—the lack of peace, while in our research the edges signify a lack of cooperation. Thus, Richardson’s conclusions can be at least partially extrapolated to municipalities. The main difference lies in the fact that people die during wars, while the lack of cooperation between municipalities slows down regional economic development. However, it may be concluded that logical homologies identified with use of the above method indicate a relatively new and interesting direction of regional research. Inter-municipal cooperation does not have to be treated as either there or not, but it can be gradable.

Richardson noted that administratively designated internal borders usually look very different from the natural external borders that separate countries. Usually, internal borders take the form of straight lines that are clearly and directly drawn on the maps, typically regardless of the natural terrain. In contrast, external borders often use natural features such as rivers or mountain ranges. His conclusions regarding the internal and external borders do not match the Warmia and Mazury Province. Very often municipal boundary lines use the natural terrain and run along rivers, lakes, forests and arable fields. Probably such a division was intended to avoid competition for the share of the same resources. On the other hand, the international border, which separates the examined province from the Kaliningrad Region, which belongs to Russia, has a much simpler course. Since the administrative lines separating the municipalities have such a complicated track, it means that the municipalities are fractals, i.e., objects showing statistical self-similarity. This is because they contain reduced copies of themselves. Therefore, the fractal dimension should be used to describe the boundaries between municipalities, not the usual length. In this way, complexity is manifested in the four-colour theorem. Therefore, this theorem can be used to colour each map, including the infinitely complex fractal patterns drawn on the complex plane [36].

## 11. Conclusions

Changes in growth poles and development axes in the studied province are represented by empirical dual graphs, which were determined for 2009, 2012, 2015 and 2019. The measure of development consists in comparing information contained in empirical graphs with the model dual graph, which was created on the basis of a map corresponding to the conditions of the four-colour theorem. Graph edges are interpreted as barriers to cooperation between neighbouring municipalities. The reference graph has the maximum number of edges, and therefore represents the case of a complete absence of growth poles and development axes. It plays the role of the absolute reference system in the research. As demonstrated, the public administration sector at the local level is very sensitive to changes in financing, which can be seen especially in the example of the implementation of telecommunications technologies. Most projects in this sector depend on the level of government spending. In the thermoeconomics terms, changes in empirical dual graphs can be treated as a result of the flow of monetary entropy through the Warmia and Mazury Province [115]. The studied region is a dissipative system that intakes low entropy from the environment, which increases the level of organisation of municipal public administration offices. This is reflected in the transition of websites to higher quality classes. Money allocated for the development of telecommunication infrastructure is the medium of low entropy. Lack of money is associated with a decrease in the quality of websites and their stabilisation in the lower classes. In this way entropy increases in the whole system. The inflow of money reduces the internal entropy of the system, which is associated with the formation of new growth poles and development axes. Maintenance of existing infrastructure at the 2012 level is therefore conditioned by the inflow of additional funds. Province authorities are well aware of this, which is why they launched the Digital Plan 2025 for Warmia and Mazury. The goal of the programme is the proper operation of the fibre-optic backbone distribution network, and thus providing access to the network to the end subscribers.

The proposed measure of economic growth contains in one number a rich collection of economic and spatial information. It includes Hicks’ condition for top technique because it incorporates wikinomics business models such as participation platforms and prosumption that could not exist without computer networks. Additionally, this measure enables the location of regional growth poles and development axes and considers the spatial diversity of municipalities and barriers in their mutual cooperation. It is based on digital technologies, so it is not limited to one industry, but covers all those industries that are controllable by software. Therefore, this measure has an advantage over the location quotient and all other classic measures of regional growth and development. Furthermore, it should be emphasised that the new measure applies to the developing economy, in which the share of high and very high quality municipal websites is significant. The four-colour theorem can be used as a reference system for empirical studies only if it corresponds to the Thom classification theorem. This signifies adequate representation of elementary catastrophes by the quality classes of the municipal websites. The proposed measure also takes into account the spatial complexity of municipalities, because in the Warmia and Mazury Province, most of them are fractals. It follows that both the management of the entire province and individual municipalities is a complex problem and only fractal organisations can cope with it [116,117,118]. Therefore, all public administration reforms should aim at giving the administration fractal features, which applies in particular to municipal public administration offices as basic units of the local government.

## Figures and Tables

**Table 1:** Criteria for the functionality of the municipal websites in 2009, 2012, 2015 and 2019.

Code	Website Functionality Criteria
A01	Website is updated on a regular basis
A02	The postal address of the office is included, directions are provided
A03	The office publishes chat lines and/or discussion lists for the citizens
A04	The structure of the office has been posted
A05	Current information is published on a regular basis
A06	There is a possibility to search for necessary information
A07	A calendar of posts is published
A08	The user can fill and send a form online
A09	Other than Polish language versions are available
A10	Website provides icons that help the user to use the website
A11	The website address of the office is intuitive
A12	Archive exists
A13	A map of the municipality is published
A14	Tourist attractions are indicated
A15	Link to “digital office” provided
A16	Link to ePUAP (Electronic Platform of Public Administration Services) provided

**Table 2:** Four clusters representing the wikinomics categories of quality of the websites of the municipal administrative authorities in 2009, 2012, 2015, and 2019.

Characteristics	Low Quality	Average Quality	High Quality	Very High Quality
**Year**	**2009**			
Points (min–max)	0–4	5–9	10–11	12–16
Centroid	0	7.72	10.45	13.35
Number of municipalities	17	22	37	40
**Year**	**2012**			
Points (min–max)	0–6	7–10	11–13	14–16
Centroid	0	9	11.75	14.45
Number of municipalities	6	11	49	50
**Year**	**2015**			
Points (min–max)	0–6	7–11	12–13	14–16
Centroid	0.47	8.69	11.75	14.65
Number of municipalities	10	32	35	39
**Year**	**2019**			
Points (min–max)	0–1	8–11	12	13–15
Centroid	1	10.46	12.00	13.87
Number of municipalities	3	30	17	66

**Table 3:** Characteristics of dual graphs corresponding to the political map of South America, the reference map and four empirical maps of the Warmia and Mazury Province.

Type of Dual Graph	Number of Vertices (*V*)	Number of Faces (*F*)	Cauchy’s Formula Interpretation	
			Number of Edges (E=V+F−1)	Euler Characteristic (χ(G)=V−E+F)
Political map of South America	13	13	25	1
The reference graph (four-colour theorem)	116	162	277	
The empirical graph 2009	36	28	63	
The empirical graph 2012	28	22	49	
The empirical graph 2015	55	52	106	
The empirical graph 2019	36	23	58	

## Data Availability

The data are not publicly available due to commercial restrictions.
